# Ring Opening Reactions through C-O Bond Cleavage Uniquely Adding Chemical Functionality to Boron Subphthalocyanine

**DOI:** 10.3390/molecules201018237

**Published:** 2015-10-07

**Authors:** Catherine Bonnier, Timothy P. Bender

**Affiliations:** 1 Department of Chemical Engineering and Applied Chemistry, University of Toronto, 200 College St., Toronto M5S 3E5, ON, Canada; 2 Department of Materials Science and Engineering, University of Toronto, 184 College St., Toronto M5S 3E4, ON, Canada; 3 Department of Chemistry, University of Toronto, 80 St. George St., Toronto M5S 3H6, ON, Canada

**Keywords:** boron, subphthalocyanine, alkoxy, alkyl, bromide, endocyclic, carbon, oxygen, bond

## Abstract

We are reporting the unexpected reaction between bromo-boron subphthalocyanine (Br-BsubPc) and THF, 1,4-dioxane or γ-butyrolactone that results in the ring opening of the solvent and its addition into the BsubPc moiety. Under heating, the endocyclic C-O bond of the solvent is cleaved and the corresponding bromoalkoxy-BsubPc derivative is obtained. These novel alkoxy-BsubPc derivatives have remaining alkyl-bromides suitable for further functionalization. The alkoxy-BsubPcs maintain the characteristic strongly absorption in visible spectrum and their fluorescence quantum yields.

## 1. Introduction

Boron subphthalocyanines (BsubPcs) [1] are a family of aromatic π-electron systems that possess several desirable features, which make them suitable in a variety of applications, including within the organic electronics field [2], of highest interest to our group is the application of BsubPc in organic photovoltaics [3,4,5,6,7,8,9,10]. The unusual bowl shape of a BsubPc is attractive due to its low tendency to aggregate in solution [11]. In addition, BsubPcs strongly absorbs visible light and possesses good to moderate fluorescence quantum yields [2].

Boron acts as an atomic template to form the subPc macrocycle during the cyclotrimerization of phthalonitrile [12]. Thereafter the boron atom has limited electronic connectivity to the molecular orbitals of the π system, indicated by the retention of most properties of BsubPcs upon varying the nature of the axial substituent [13]. The physicochemical properties of BsubPcs can be most significantly tuned by substitution of hydrogen atoms around the periphery of the subPc macrocyle. However, the solid-state packing of BsubPcs has been found to be highly dependent on the nature of the axial substituent, leading to the synthesis and crystallographic analyses of derivatives possessing a variety of ligands, such as phenoxy [14], sulfonates [15], and halides [16]. 

As part of an ongoing research project in our laboratory, we have previously optimized the synthesis of halo-BsubPcs (halo = F, Cl, Br) and investigate their properties [16]. It was found that the stability of halo-BsubPcs towards hydrolysis and phenoxylation follows the trend F > Cl > Br, suggesting that Br-BsubPc would be a promising starting/precursor material to access a variety of axial-substituted BsubPc derivatives due to its highest reactivity. Conversely, the enhanced reactivity of Br-BsubPc may also increase the probability of generating by-products, suggesting that modifying or optimizing of the reaction conditions is likely to be critical. 

**Scheme 1:**
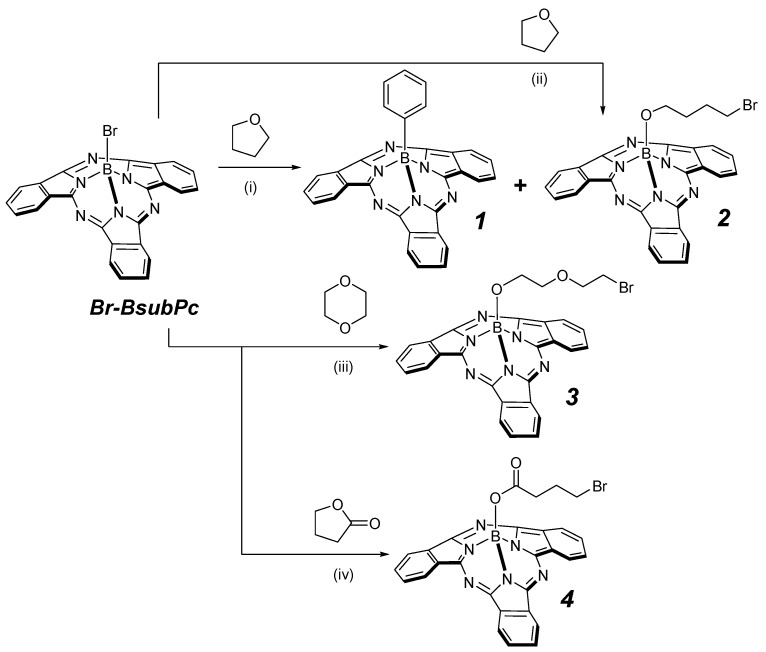
Reaction of Br-BsubPc (**1**) to yield ring opened products with terminal alkyl bromides in presence of solvents bearing an endocyclic C-O bond. Conditions: (i) PhMgBr, THF reflux; (ii) 2,4,6-trifluorophenyl-MgBr, THF, reflux; (iii) 1,4-Dioxane, reflux; and (iv) γ-Butyrolactone, reflux.

We have recently been investigating the synthesis and application of phenylated BsubPcs [17,18,19,20]. During our study we saw only a moderate yield in the reaction of Br-BsubPc with phenyl-MgBr (Scheme 1). The resulting phenyl-BsubPc was found to have application in photovoltaic cells [21]. However, during this study we also identified an interesting side product during the attempted reaction of fluorophenyl Grignard reagents with Br-BsubPc (Scheme 1). Herein we report the detailed analysis of this side reaction, propose a mechanism for the reaction and explore the scope of the reaction that yielded the first examples of the reaction of Br-BsubPc with standard solvent molecules bearing an endocyclic C-O bond. The novel alkoxy-BsubPc derivatives have potential application as synthetic intermediates owing to the presence of an alkyl bromide in the axial position.

## 2. Results and Discussion

In our attempts to prepare BsubPc derivatives for organic electronic applications, we have explored Br-BsubPc as a starting material to react with various aryl Grignard reagents in anhydrous THF under reflux conditions (Scheme 1) [21]. After 16 h, HPLC analysis of the crude reaction mixture showed that, in addition to the desired axially-substituted aryl-BsubPc product (**1**), an impurity (**2**) was present in a significant quantity (>30%). Moreover, upon refluxing 2,4,6-trifluorophenyl magnesium bromide with Br-BsubPc in THF for four days under an atmosphere of argon (Scheme 1), by-product **2** was the only species bearing a BsubPc fragment to be detected. Isolation of **2** from this reaction mixture was achieved by column chromatography (see Supporting Material).

The ^1^H-NMR spectrum of **2** clearly indicates the presence of a symmetrical BsubPc macrocycle, indicating that substitution at the axial position occurred. Signals associated with the desired axial aryl fragment were absent; instead, an internal butyl chain with absence of a triplet at high fields was observed (see Supporting Material). Mass spectrometry confirmed the presence of an oxygen and bromine atom. Put together, these observations indicated that the identity of the material **2** was 4-bromobutoxy-BsubPc. The formation of 4-bromobutoxy-BsubPc could only have resulted from the ring-opening of the THF solvent.

The absence of the 2,4,6-trifluorophenyl moiety in **2** suggests that the nucleophilicity of the Grignard reagent was too low to react with Br-BsubPc, and as a consequence, Br-BsubPc may have simply reacted with THF due to the long reaction time (three days). We therefore took Br-BsubPc and refluxed it alone in THF under an argon atmosphere and the reaction was monitored by HPLC. After two days, the purple suspension turned green-yellow in color and thereafter no BsubPc was detected. We, therefore, concluded that in this particular case, the presence of a Grignard reagent is necessary to promote the C-O bond cleavage.

Conversely, we took other cyclic solvents containing an endocyclic C-O bond and tested then alone in the presence of Br-BsubPc. For example, Br-BsubPc was heated to reflux in the presence of 1,4-dioxane and γ-butyrolactone, cleanly affording (2-(2-bromoethoxy)ethoxy)-BsubPc **3** and 4-bromobutanoyl-BsubPc **4**, respectively in the absence of any Grignard reagent (Scheme 1). In the latter, the absence of a signal at *ca.* δ = 65 ppm in the ^13^C-NMR spectrum [18] confirmed that the sp^3^-hybrized carbon atom of γ-butyrolactone was involved in the bond cleavage (see Supporting Material). The use of acyclic ethers such as diethyl ether showed no conversion, suggesting that a cyclic solvent molecule is necessary to observe C-O bond cleavage.

Aside from ring-opening endocyclic C-O bonds, the reactivity of cyclic solvents containing other heteroatoms than oxygen was investigated. Upon heating Br-BsubPc under an atmosphere of argon in presence of *N*-methyl-2-pyrrolidinone, the nitrogen analog of γ-butyrolactone, partial hydrolysis of Br-BsubPc occurred, affording hydroxy-BsubPc (identified by HPLC analysis against a standard); similar results were observed when using hexamethylcyclotrisiloxane. In the presence of two different heteroatoms such as in morpholine, decomposition of the BsubPc macrocycle is observed, even at room temperature. These observations suggest that the reported reaction is very selective and occurs in presence of solvents containing an endocyclic C-O bond only.

**Scheme 2:**
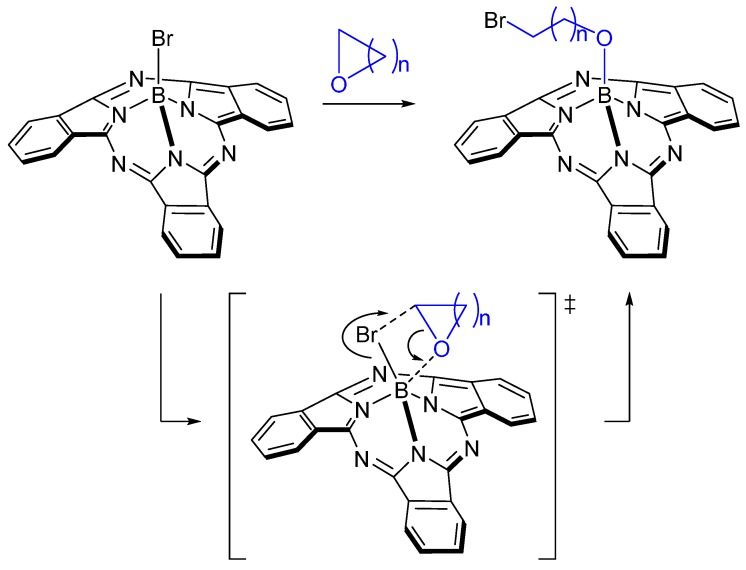
Proposed mechanism for the formation of BsubPcs **2**–**4**.

The cleavage of the C-O bond and ring-opening of THF has been reported to occur in presence of strong Brønsted acids, such as anhydrous HBr [22] or tricoordinated boron Lewis acidic centers [23,24]. Clearly, the mechanism of ring-opening involving Br-BsubPc is different due to the lack of protic and/or Lewis acidic components. We hypothesize that the weak B-Br bond of Br-BsubPc is solely responsible for facilitating this reaction, and the nature of the leaving group does not play a significant role. This is consistent with the absence of by-product formed when triflate-BsubPc is prepared in THF [25]. While recent theoretical studies have looked at the corresponding radical and cation of BsubPc when the B-X bond is cleaved [26,27], the proposed formation of a four-membered ring complex intermediate by interaction of a nucleophilic oxygen centre (Scheme 2) is consistent with the recent findings of Guilleme *et al.* [28]. We then hypothesize that given enough ring strain within the cyclic ether the initial complexation is followed by σ-bond metathesis (Scheme 2) yielding BsubPcs 2-4, with the bromide being exclusively inserted at the α position of the oxygen heteroatom yielding an alkyl bromide at the ω-position of the axial substituent. Why or how the reaction with THF, a cyclic ether with limited ring strain, needs the presence of a Grignard reagent is unclear.

**Figure 1:**
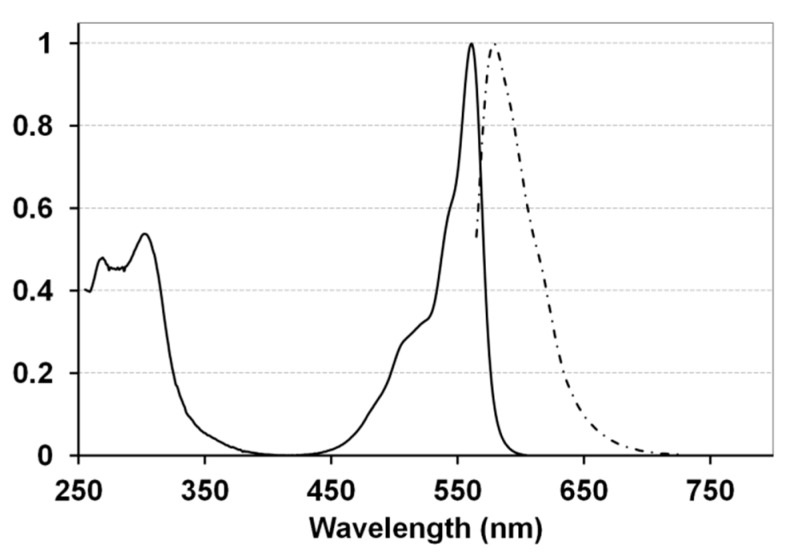
Normalized absorption (—) and fluorescent emission (–·–·–, excitation at 561 nm) spectra of **2** recorded in CH_2_Cl_2_ solution.

BsubPc derivatives **2**–**4**, bearing a bromoalkoxy chain at the axial position will unlikely be suitable for application in organic electronic devices as the presence of an *n-*alkyl chain makes them unstable during vacuum sublimation. Furthermore their amorphous nature (*i.e.*, lack of tendency to crystallize) will further limit their application in organic electronic devices. However, we feel they may have application as solution deposited functional materials or as singlet oxygen generators for photodynamic therapy likely as synthetic intermediates. Moreover, the presence of a terminal halide in **2**–**4** offers the possibility to access a more complex library of biological relevant BsubPc derivatives via further derivatization through the bromide leaving group. For example, Xu *et al.* reported cytotoxic agents based on axial oligoethylene glycol BsubPcs, prepared upon reacting the appropriate alcohol precursor with Cl-BsubPc in very low yields (<10%) [29]. The bromo alkylated BsubPc described herein are obtained in relatively good yields and are strongly absorbing in the visible light region (Figure 1, ε of Q band > 50 000 M^−1^∙cm^−1^) and have fluorescence quantum yields of *ca.* 0.57 properties (Table 1) desirable for the above mentioned applications including chemical functionality. 

**Table 1:** Physicochemical properties of BsubPcs **2**–**4** ^a^.

	Q band		Soret Band		Fluorescence	
	λ_max_ (nm)	Log ε	λ_max_ (nm)	Log ε	λ_max_ (nm)	ϕ_FL_^b^
**2**	561	4.73	302	4.46	579	0.44 ± 0.05
**3**	561	4.94	303	4.61	581	0.45 ± 0.03
**4**	564	4.96	304	4.63	585	0.45 ± 0.04

^a^ Recorded in CH_2_Cl_2_; ^b^ Measured in deoxygenated CH_2_Cl_2_ using phenoxy-dodecafluoroBsubPc as the standard (ϕ_FL_ = 0.40) [30] according to the methodology previously described [31].

## 3. Experimental Section

### 3.1. General Considerations

Hexanes, EtOAc, THF and 1,4-dioxane, and HPLC grade acetonitrile and *N*,*N*-dimethylformamide solvents were purchased from Caledon Laboratory Ltd. (Caledon, ON, Canada); THF was dried and purified by passing through activated alumina and 1,4-dioxane was dried over 4Å molecular sieves. Mg turnings, I_2_ and γ-butyrolactone were purchased from Sigma Aldrich (Mississauga, ON, Canada) and used as is. Bromo-2,4,6-trofluorobenzene was purchased from Oakwood Products Inc. (West Columbia, SC, USA) and used as is. Column chromatography was carried out on Silicycle silica 60 silica gel (particle size 40–63 μm) (Quebec City, QC, Canada) and thin layer chromatography on silica gel 60 coated with F_254 nm_. All NMR spectra were recorded in CDCl_3_ (purchased from Cambridge Isotope Laboratories Inc. (Tewksbury, MA, USA) and used as is) on a Bruker Advance III 400 MHz spectrometer operating at 400 MHz (^1^H), 128 MHz (^11^B), and 100 MHz (^13^C) at 25 °C. Chemical shifts are reported in ppm relative to the residual solvent signal (^1^H and ^13^C) and BF_3_•OEt_2_ (^11^B, 0 ppm) standard. High-pressure liquid chromatography analyses were carried on a Waters 2695 separation module with a Waters 2998 photodiode array and a Waters Styragel HR 2 THF 4.6 × 300 mm column. The mobile phase consisted of MeCN:DMF (4:1 *v*/*v*). UV-VIS spectra were obtained using a PerkinElmer Lambda 25 spectrophotometer operating in double-beam mode with a slit width of 1 nm. Fluorescence spectra were obtained using a PerkinElmer LS55 spectrophotometer. All reactions were carried out in a standard fumehood under ambient lighting conditions.

Br-BsubPc (**1**) was synthesized according to the literature procedure [16].

### 3.2. Syntheses

*4-Bromobutoxy-BsubPc* **2**. Under an atmosphere of Ar, magnesium turnings (0.288 g, 12 mmol) and a crystal of I_2_ were added to 15 mL of anhydrous THF. The slurry was stirred until complete dissolution of I_2_ and 1-bromo-2,4,6-trifluorobenzene (1.18 mL, 10 mmol) was added dropwise and the mixture was stirred 1.5 h at room temperature. The solution was then transferred to a suspension of Br-BsubPc (2.447 g, 5.15 mmol) in 400 mL of anhydrous THF. The mixture was refluxed for four days and after being cooled to room temperature, volatiles were removed via rotovap. The crude material was purified by column chromatography over SiO_2_ using hexanes:EtOAc (gradient from 3:1 to 1:1) as the eluent. The oily material was triturated MeOH and the solids were filtered and dried, affording **2** as a magenta solid (0.356 g, 0.76 mmol, 15%). ^1^H-NMR (400 MHz, CDCl_3_): δ = 8.85 (m, 6H), 7.90 (m, 6H), 2.90 (t, 2H, ^3^J_HH_ = 6.6 Hz), 1.67 (br, 1H), 1.48 (t, 2H, ^3^J_HH_ = 6.4 Hz), 1.07 (m, 2H), 0.64 (m, 2H). ^13^C{^1^H}-NMR (100 MHz, CDCl_3_): δ = 151.6, 131.1, 129.9, 122.2, 58.2, 33.5, 29.4, 28.9. ^11^B{^1^H}-NMR (128 MHz, CDCl_3_): δ = −14.8 (s). HRMS (DART): [M + H]^+^ *m*/*z* calcd [^12^C_28_^1^H_21_^11^B^79^Br^14^N_6_^16^O]^+^ 547.10533; found, 547.10603.

*2-(2-Bromoethoxy)ethoxy-BsubPc* **3**. Under an atmosphere of Ar, Br-BsubPc (500 mg, 1.05 mmol) was suspended in 60 mL of dry 1,4-dioxane and the mixture was refluxed three days. Volatiles were removed *in vacuo* and the residue was purified via column chromatography over SiO_2_ using hexanes:EtOAc 2:1 as the eluent, affording **3** as a magenta solid (0.161 g, 0.29 mmol, 27%). ^1^H-NMR (400 MHz, CDCl_3_): δ = 8.85 (m, 6H), 7.90 (m, 6H), 3.19 (t, 2H, ^3^J_HH_ = 6.4 Hz), 3.03 (t, 2H), 2.58 (t, 2H, ^3^J_HH_ = 4.9 Hz), 1.64 (t, 2H). ^13^C{^1^H}-NMR (100 MHz, CDCl_3_): δ = 151.6, 131.1, 129.8, 122.2, 71.1, 70.8, 58.8, 30.0. ^11^B{^1^H}-NMR (128 MHz, CDCl_3_): δ = −14.7 (s). HRMS (DART): *m*/*z* calcd [^12^C_28_^1^H_21_^11^B^79^Br^14^N_6_^16^O_2_]^+^ 563.10024; found, 563.10017.

*4-Bromobutanoyl-BSubPc* **4**. Under an atmosphere of Ar, Br-BsubPc (500 mg, 1.05 mmol) was suspended in 60 mL of γ-butyrolactone and the mixture was refluxed 12 h. Volatiles were removed *in vacuo* and the residue was purified via column chromatography over SiO_2_ using hexanes:EtOAc 3:1 as the eluent, affording **4** as a magenta solid (0.185 g, 0.33 mmol, 31%). ^1^H-NMR (400 MHz, CDCl_3_): δ = 8.88 (m, 6H), 7.92 (m, 6H), 2.95 (m, 2H), 1.45 (m, 4H). ^13^C{^1^H}-NMR (100 MHz, CDCl_3_): δ = 171.3, 151.7, 131.1, 130.0, 122.4, 32.8, 32.7, 27.1. ^11^B{^1^H}-NMR (128 MHz, CDCl_3_): δ = −15.2 (s). HRMS (DART): [M + H]^+^ *m*/*z* calcd [^12^C_28_^1^H_19_^11^B^79^Br^14^N_6_^16^O_2_]^+^ 561.08459; found, 561.08300.

## 4. Conclusion

The ring-opening reaction of solvents containing an endocyclic C-O bond facilitated by Br-BsubPc and yielding bromo alkylated BsubPc was reported, its scope was probed and the potential use of these novel BsubPc has been outlined. These novel derivatives possess strong absorptions in the visible region with good fluorescence quantum yields, despite the presence of a heavy bromine atom. The new alkoxy-BsubPcs could be suitable for biological applications or as synthons to access more complex BsubPc derivatives.

## Supplementary Material

Supplementary data associated with this article (general methods, synthesis, absorption, fluorescence, ^1^H- and ^13^C{^1^H}-NMR spectra) can be found in the online version. Supplementary materials can be accessed at: http://www.mdpi.com/1420-3049/20/10/18237/s1.
