# Supplementary material for: Ring Opening Reactions through C-O Bond Cleavage Uniquely Adding Chemical Functionality to Boron Subphthalocyanine
Source: Molecules. 2015 Oct 7;20(10):18237–45. doi: 10.3390/molecules201018237 (PMC6332161; doi:10.3390/molecules201018237)
Supplement: Supplementary file 1 [file molecules-20-18237-s001.pdf]

# Supporting Information

*General Considerations*

Hexanes, EtOAc, THF and 1,4-dioxane, and HPLC grade acetonitrile and *N*,*N*-dimethylformamide solvents were purchased from Caledon Laboratory Ltd. (Caledon, ON, Canada); THF was dried and purified by passing through activated alumina and 1,4-dioxane was dried over 4Å molecular sieves. Mg turnings, $I_2$ and γ-butyrolactone were purchased from Sigma Aldrich (Mississauga, ON, Canada) and used as is. Bromo-2,4,6-trofluorobenzene was purchased from Oakwood Products Inc. (West Columbia, SC, USA) and used as is. Column chromatography was carried out on Silicycle silica 60 silica gel (particle size 40–63 μm) (Quebec City, Quebec, Canada) and thin layer chromatography on silica gel 60 coated with $F_{254\ nm}$. All NMR spectra were recorded in $CDCl_3$ (purchased from Cambridge Isotope Laboratories Inc. (Tewksbury, MA, USA) and used as is) on a Bruker Advance III 400 MHz spectrometer operating at 400 MHz ($^1$H), 128 MHz ($^{11}$B), and 100 MHz ($^{13}$C) at 25 °C. Chemical shifts are reported in ppm relative to the residual solvent signal ($^1$H and $^{13}$C) and $BF_3$•$OEt_2$ ($^{11}$B, 0 ppm) standard. High-pressure liquid chromatography analyses were carried on a Waters 2695 separation module with a Waters 2998 photodiode array and a Waters Styragel HR 2 THF 4.6 × 300 mm column. The mobile phase consisted of MeCN:DMF (4:1 *v*/*v*). UV-VIS spectra were obtained using a PerkinElmer Lambda 25 spectrophotometer operating in double-beam mode with a slit width of 1 nm. Fluorescence spectra were obtained using a PerkinElmer LS55 spectrophotometer. All reactions were carried out in a standard fumehood under ambient lighting conditions.

Br-BsubPc (**1**) was synthesized according to the literature procedure [1].

*Syntheses*

*4-Bromobutoxy-BsubPc* **2**. Under an atmosphere of Ar, magnesium turnings (0.288 g, 12 mmol) and a crystal of $I_2$ were added to 15 mL of anhydrous THF. The slurry was stirred until complete dissolution of $I_2$ and 1-bromo-2,4,6-trifluorobenzene (1.18 mL, 10 mmol) was added dropwise and the mixture was stirred 1.5 h at room temperature. The solution was then transferred to a suspension of Br-BsubPc (2.447 g, 5.15 mmol) in 400 mL of anhydrous THF. The mixture was refluxed for four days and after being cooled to room temperature, volatiles were removed via rotovap. The crude material was purified by column chromatography over $SiO_2$ using hexanes:EtOAc (gradient from 3:1 to 1:1) as the eluent. The oily material was triturated MeOH and the solids were filtered and dried, affording **2** as a magenta solid (0.356 g, 0.76 mmol, 15%). $^1$H-NMR (400 MHz, $CDCl_3$): δ = 8.85 (m, 6H), 7.90 (m, 6H), 2.90 (t, 2H, $^3J_{HH}$ = 6.6 Hz), 1.67 (br, 1H), 1.48 (t, 2H, $^3J_{HH}$ = 6.4 Hz), 1.07 (m, 2H), 0.64 (m, 2H). $^{13}$C{$^1$H}-NMR (100 MHz, $CDCl_3$): δ = 151.6, 131.1, 129.9, 122.2, 58.2, 33.5, 29.4, 28.9. $^{11}$B{$^1$H}-NMR (128 MHz, $CDCl_3$): δ = −14.8 (s). HRMS (DART): $[M + H]^+$ *m*/*z* calcd $[^{12}C_{28}{}^1H_{21}{}^{11}B^{79}Br^{14}N_6{}^{16}O]^+$ 547.10533; found, 547.10603.

*2-(2-Bromoethoxy)ethoxy-BsubPc* **3**. Under an atmosphere of Ar, Br-BsubPc (500 mg, 1.05 mmol) was suspended in 60 mL of dry 1,4-dioxane and the mixture was refluxed three days. Volatiles were removed *in vacuo* and the residue was purified via column chromatography over $SiO_2$ using hexanes:EtOAc 2:1 as the eluent, affording **3** as a magenta solid (0.161 g, 0.29 mmol, 27%). $^1$H-NMR (400 MHz, $CDCl_3$): δ = 8.85 (m, 6H), 7.90 (m, 6H), 3.19 (t, 2H, $^3J_{HH}$ = 6.4 Hz), 3.03 (t, 2H), 2.58 (t, 2H, $^3J_{HH}$ = 4.9 Hz), 1.64 (t,

2H). $^{13}C\{^1H\}$-NMR (100 MHz, $CDCl_3$): δ = 151.6, 131.1, 129.8, 122.2, 71.1, 70.8, 58.8, 30.0. $^{11}B\{^1H\}$-NMR (128 MHz, $CDCl_3$): δ = −14.7 (s). HRMS (DART): *m*/*z* calcd $[^{12}C_{28}{}^1H_{21}{}^{11}B^{79}Br^{14}N_6{}^{16}O_2]^+$ 563.10024; found, 563.10017.

*4-Bromobutanoyl-BSubPc* **4**. Under an atmosphere of Ar, Br-BsubPc (500 mg, 1.05 mmol) was suspended in 60 mL of γ-butyrolactone and the mixture was refluxed 12 h. Volatiles were removed *in vacuo* and the residue was purified via column chromatography over $SiO_2$ using hexanes:EtOAc 3:1 as the eluent, affording **4** as a magenta solid (0.185 g, 0.33 mmol, 31%). $^1H$-NMR (400 MHz, $CDCl_3$): δ = 8.88 (m, 6H), 7.92 (m, 6H), 2.95 (m, 2H), 1.45 (m, 4H). $^{13}C\{^1H\}$-NMR (100 MHz, $CDCl_3$): δ = 171.3, 151.7, 131.1, 130.0, 122.4, 32.8, 32.7, 27.1. $^{11}B\{^1H\}$-NMR (128 MHz, $CDCl_3$): δ = −15.2 (s). HRMS (DART): $[M + H]^+$ *m*/*z* calcd $[^{12}C_{28}{}^1H_{19}{}^{11}B^{79}Br^{14}N_6{}^{16}O_2]^+$ 561.08459; found, 561.08300.

**Absorption and Fluorescence Spectra**

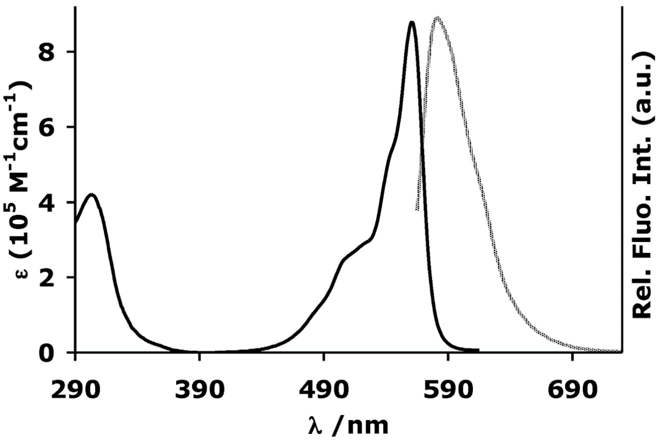


**Figure S1.** UV-vis (black) and photoluminescence (grey) spectra of **3** recorded in $CH_2Cl_2$. The excitation monochromator was set at 561 nm.

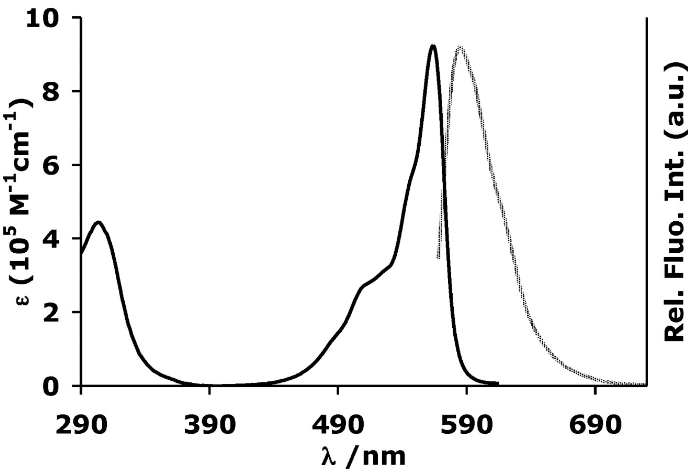


**Figure S2.** UV-vis (black) and photoluminescence (grey) spectra of **4** recorded in $CH_2Cl_2$. The excitation monochromator was set at 564 nm.

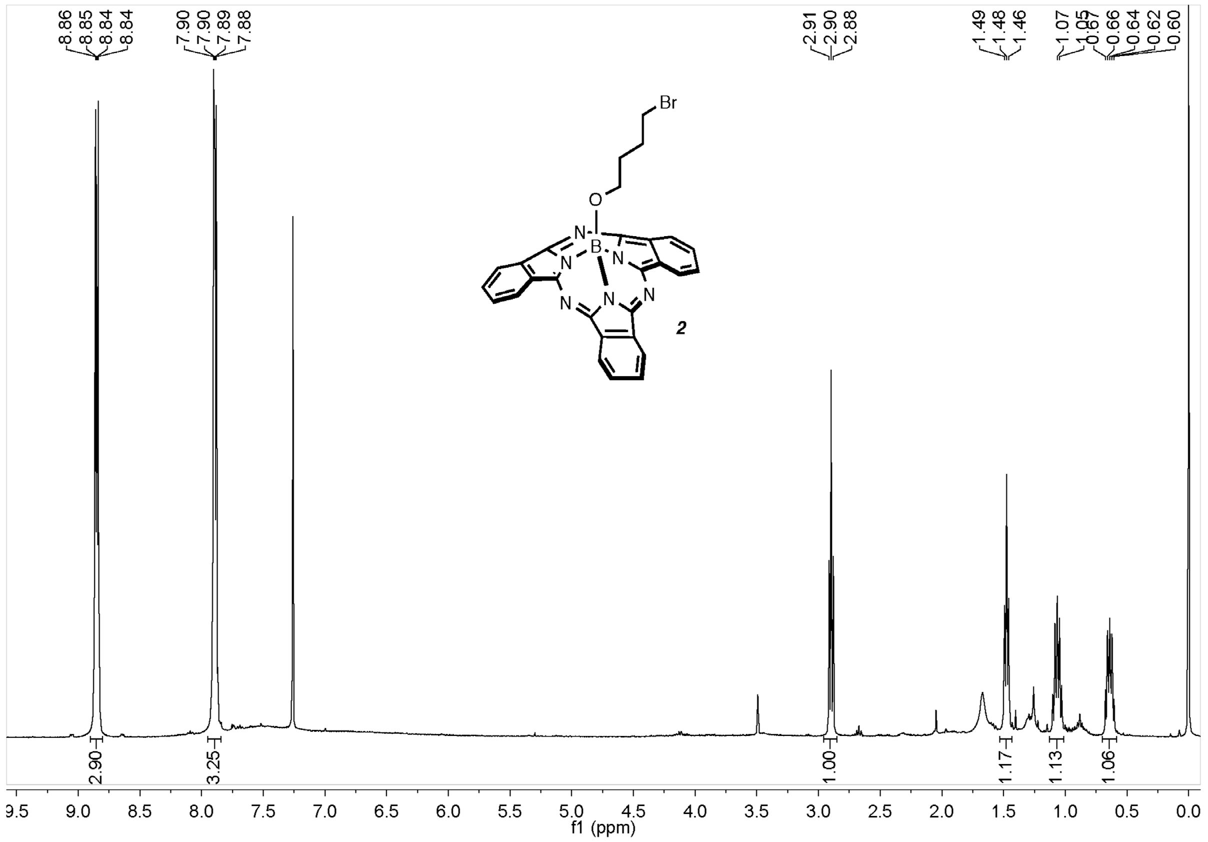


**Figure S3.** $^{1}$H-NMR spectrum of compound **2**.

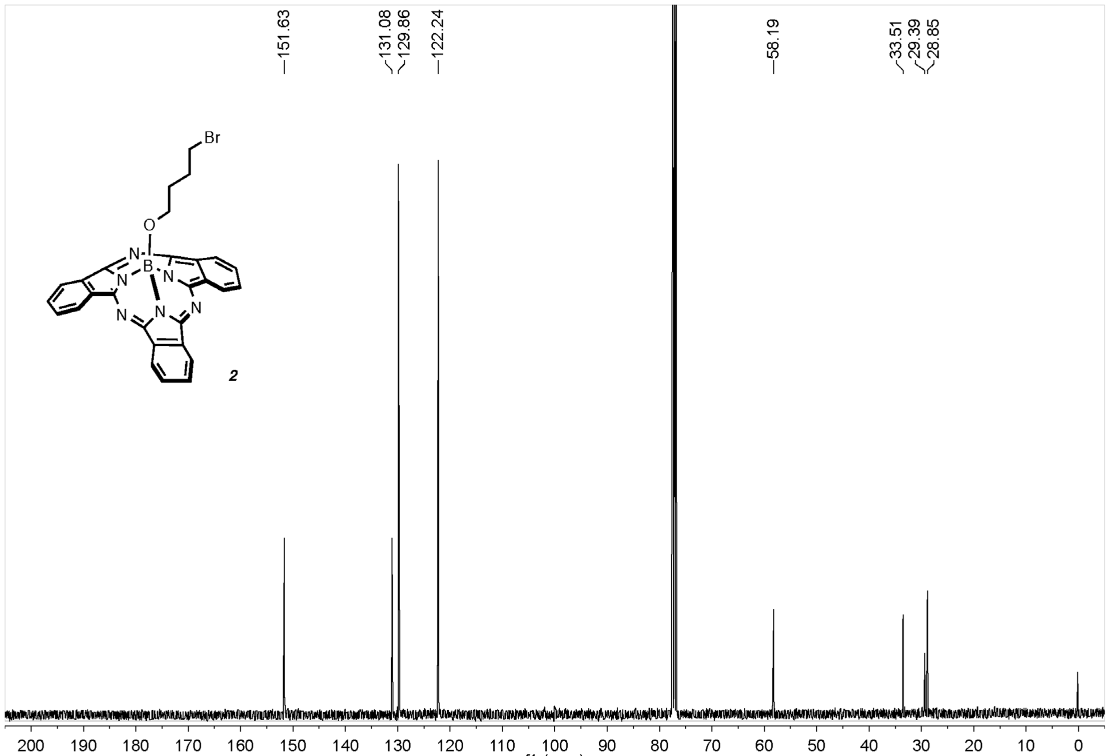


**Figure S4.** $^{13}$C-NMR spectrum of compound **2**.

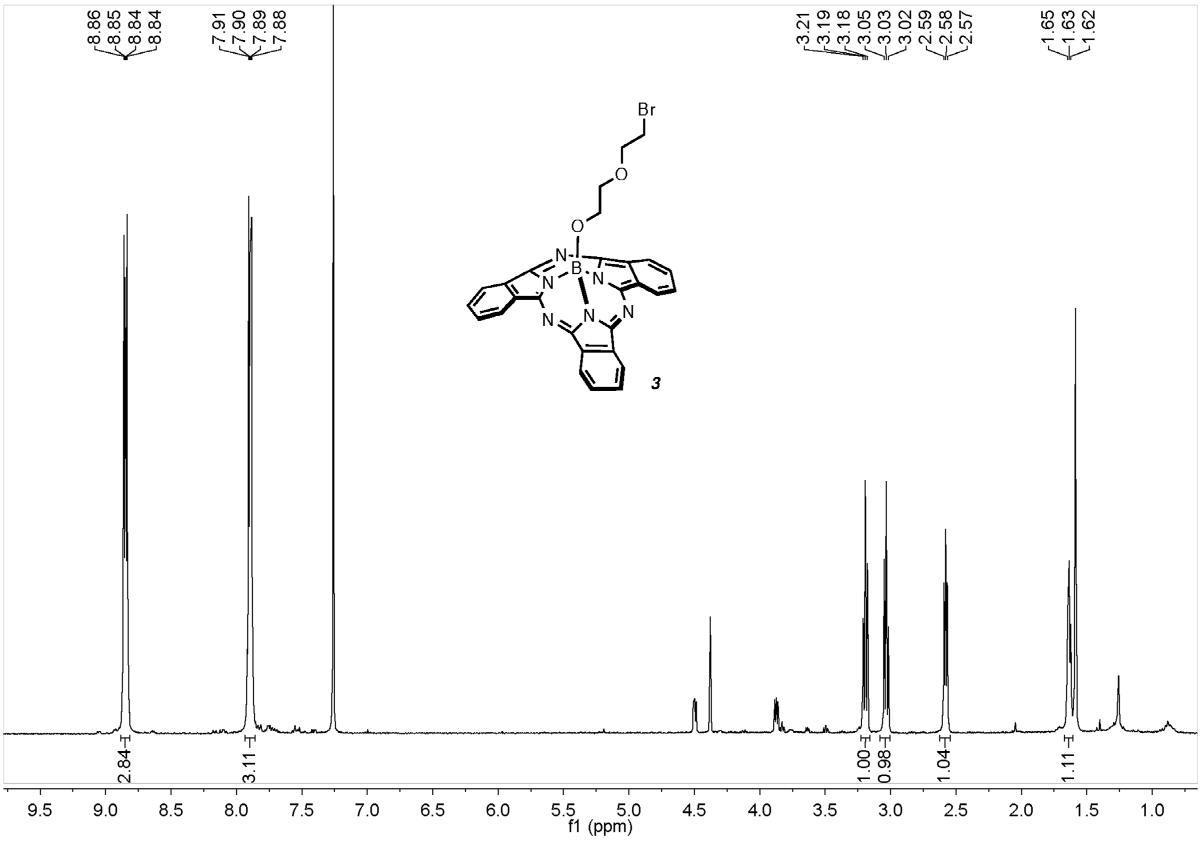


**Figure S5.** 1H-NMR spectrum of compound **3**.

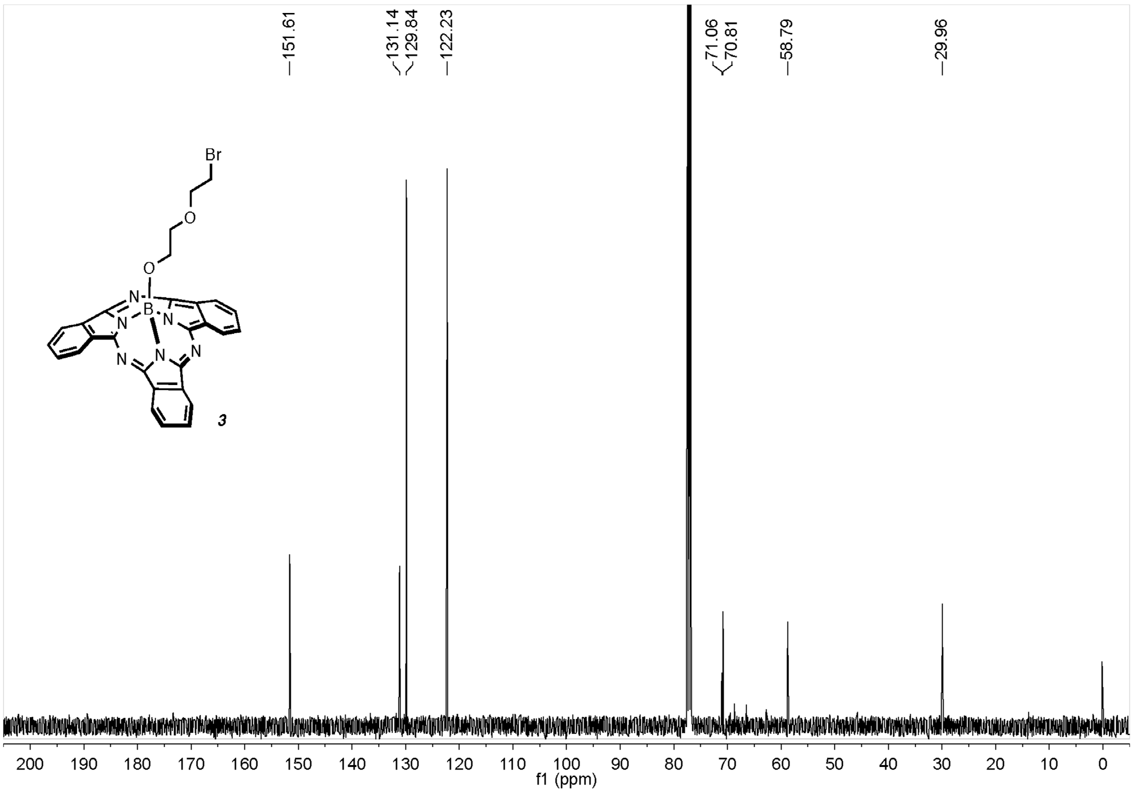


**Figure S6.** 13C NMR spectrum of compound **3**.

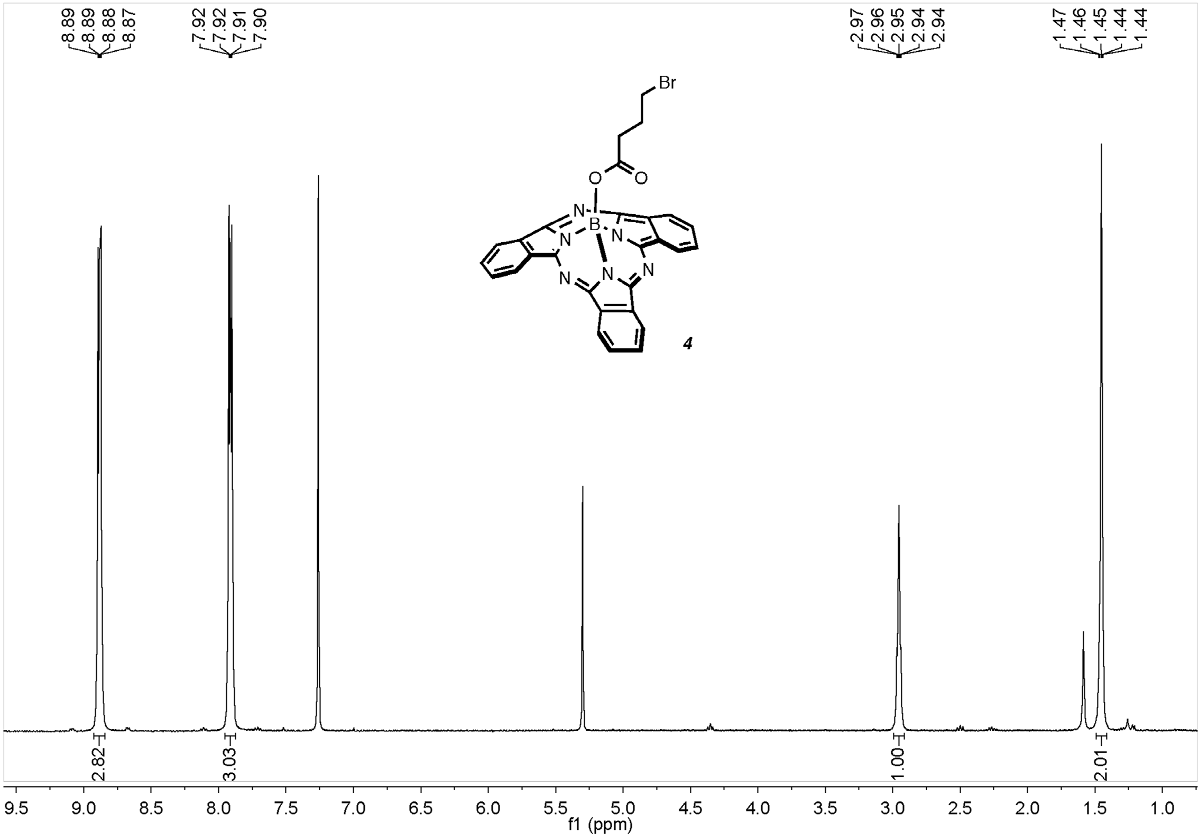


**Figure S7.** 1H-NMR spectrum of compound **4**.

## Reference

1. Fulford, M.V.; Jaidka, D.; Paton, A.S.; Morse, G.E.; Brisson, E.R.L.; Lough, A.J.; Bender, T.P. Crystal structures, reaction rates, and selected physical properties of halo-boronsubphthalocyanines (halo = fluoride, chloride, and bromide). *J. Chem. Eng. Data* **2012**, *57*, 2756–2765.
